# Increased risk of ischemic stroke associated with elevated gamma-glutamyl transferase level in adult cancer survivors: a population-based cohort study

**DOI:** 10.1038/s41598-023-43839-8

**Published:** 2023-10-06

**Authors:** Kyuwoong Kim, Hyeyun Jung, Edvige Di Giovanna, Tae Joon Jun, Young-Hak Kim

**Affiliations:** 1 National Cancer Control Institute, National Cancer Center, Goyang, Republic of Korea; 2 The Institute of Clinical Sciences, Imperial College London, London, UK; 3 Department of Diagnostic and Interventional Radiology, Ammerland-Klinik, Westerstede, Lower Saxony Germany; 4 Big Data Research Center, Asan Institute for Life Science, Asan Medical Center, Seoul, Republic of Korea; 5 Division of Cardiology, Department of Internal Medicine, Asan Medical Center, University of Ulsan College of Medicine, 88, Olympic-ro 43 gil, Songpa-gu, Seoul, 05505 Republic of Korea

**Keywords:** Medical research, Neurology, Risk factors

## Abstract

Adult cancer survivors may have an increased risk of developing ischemic stroke, potentially influenced by cancer treatment-related factors and shared risk factors with stroke. However, the association between gamma-glutamyl transferase (GGT) levels and the risk of ischemic stroke in this population remains understudied. Therefore, our study aimed to examine the relationship between GGT levels and the risk of ischemic stroke using a population-based cohort of adult cancer survivors. A population-based cohort of adult cancer survivors was derived from the National Health Insurance Service-Health Screening Cohort between 2003 and 2005 who survived after diagnosis of primary cancer and participated in the biennial national health screening program between 2009 and 2010. Cox proportional hazards model adjusted for sociodemographic factors, health status and behavior, and clinical characteristics was used to investigate the association between GGT level and ischemic stroke in adult cancer survivors. Among 3095 adult cancer survivors, 80 (2.58%) incident cases of ischemic stroke occurred over a mean follow-up of 8.2 years. Compared to the lowest GGT quartile, the hazard ratios (HRs) for ischemic stroke were 1.56 (95% CI 0.75–3.26), 2.36 (95% CI 1.12–4.99), and 2.40 (95% CI 1.05–5.46) for the second, third, and fourth sex-specific quartiles, respectively (*P*_trend_ = 0.013). No significant effect modification was observed by sex, insurance premium, and alcohol consumption. High GGT level is associated with an increased risk of ischemic stroke in adult cancer survivors independent of sex, insurance premium, and alcohol consumption.

## Introduction

Stroke continues to be a major public health concern worldwide despite remarkable progress in treatment and prevention strategies. According to *World Stroke Organization (WSO): Global Stroke Fact Sheet 2022*, the burden of stroke increased significantly with a surge in incident cases and deaths from stroke by 70% and 43% from 1990 to 2019 with the highest rates observed in low-and middle-income countries^1^. Well-established studies from the European and US cohorts have shown increased risk of stroke as compared to those who were never diagnosed with cancer, which could be attributed to both treatment-related toxicities and shared lifestyle-related risk factors^2–5^. Also, the population of cancer survivors is rapidly increasing in Europe and other parts of the world, with an estimated 12 million cancer survivors in Europe alone^6^.

The 2022 European Society of Cardiology (ESC) guidelines on cardio-oncology, developed with input from the European Hematology Association (EHA), the European Society for Therapeutic Radiology and Oncology (ESTRO), and the International Cardio-Oncology Society (IC-OS), emphasize the need for preventing cardiovascular complications in cancer survivors by addressing metabolic syndrome, abnormal lipid levels, and diabetes^7^. However, the role of serum gamma-glutamyl transferase (GGT), a liver enzyme involved in the metabolism of glutathione-S-conjugates, which has emerged as a potential biomarker for stroke^8–12^ is not well understood among adult cancer survivors. A meta-analysis of 10 prospective studies of nearly 900,000 participants showed that elevated GGT level was associated with a 28% increased risk of stroke compared to those with normal level of GGT in the general population^13^. Higher stroke risk in adult cancer survivors have been well documented among those with lifestyle-related health conditions such as diabetes and hypertension^14–16^, but there is a paucity of evidence on the association between GGT levels and stroke risk, particularly in adult cancer survivors. Given the intricate interplay of shared risk factors among patients with cancer^5^, exploring the relationship between GGT levels and the risk of stroke in adult cancer survivors would be of interest in providing valuable insights for the management of stroke risk in long-term survivorship care practices^17^.

In this study, we examined the association between GGT level and risk of developing ischemic stroke and further investigated modifying effects by socioeconomic status and alcohol consumption in a retrospective cohort of adult cancer survivors.

## Materials and methods

### Data source

We used the National Health Insurance Service-Health Screening Cohort (NHIS-HEALS) database to identify adult cancer survivors with data on GGT, ischemic stroke, and covariates^18^. The NHIS-HEALS is a dataset that includes health information on a cohort of individuals who underwent national health screening provided by the NHIS under the National Health Insurance Act in the Republic of Korea. The dataset contains information on sociodemographics, health behavior s, laboratory test results, medical claims and drug prescription records that undergo data quality-control on regular basis. Since 2002, the information on outpatient and inpatient medical claims are recorded with the *International Classification of Diseases, the 10th revision* (ICD-10) in the NHIS-HEALS. Previous studies have used the NHIS-HEALS for epidemiologic studies including neurological disorders^19–21^. Validity and details of the NHIS-HEALS database is described in detail elsewhere^22^. The study protocols strictly adhered to the Declaration of Helsinki for human subject involvement.

### Study design and population

**Figure 1:**
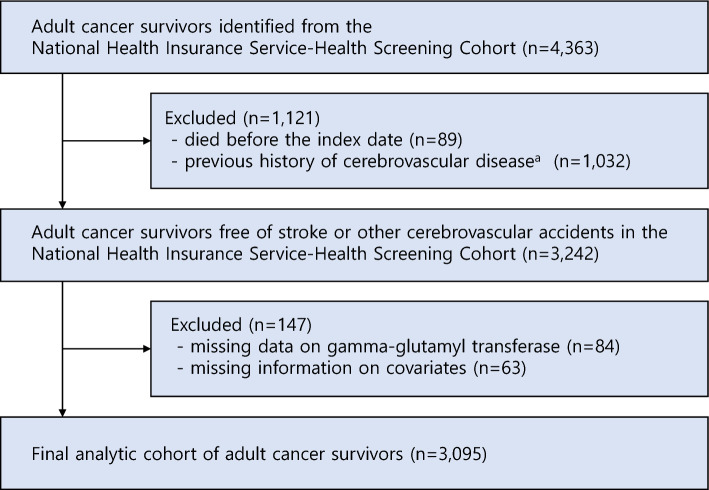
Flowchart of the study population selection from the National Health Insurance Service-Health Screening Cohort. ^a^Identified from the medical claims records with ICD-10 codes (I60–I69). *ICD-10* international classification of diseases, the tenth edition.

This study was a retrospective cohort study including adult cancer survivors who were alive at least five years post-diagnosis, in accordance with the criteria from the *American Cancer Society*^23^. Therefore, we included those who were diagnosed with cancer (ICD-10: C00–C97) from January 1, 2003 to December 31, 2005 and survived after primary cancer diagnosis and participated in the biennial national health screening program between January 1, 2009 and December 31, 2010. Due to the eligibility criteria for participating in the national health screening program of the NHIS, all participants were aged 40 years or older at the time of cohort registration in 2002^18,24^. Among 4363 adult cancer survivors who were initially identified from the NHIS-HEALS database, we excluded the participants who died before the index date (n = 89) and with previous history of cerebrovascular disease (ICD-10: I60–I69) (n = 1032). In addition, those with missing data on GGT (n = 84) and covariates (n = 63) were excluded. The final analytic cohort included 3095 adult cancer survivors (Fig. 1).

### Assessment of gamma-glutamyl transferase

As part of the National Health Screening program, participants underwent venous blood draws, and blood samples were collected for analysis using automated chemistry analyzers. This screening process allows for the detection of various biomarkers and health indicators that can be used to assess risk for a range of health conditions. Based on the blood samples, the serum GGT level was measured using the kinetic colorimetric method in a well-controlled laboratory setting, which involves the transfer of gamma-glutamyl group from GGT to a substrate, resulting in the production of a colored product that can be quantified spectrophotometrically. According to the previous study^25^, we categorized the GGT level into sex-specific quartiles (men: ≤ 20, 21–30, 31–52, ≥ 54 IU/L; women: ≤ 11, 12–15, 16–22, ≥ 23 IU/L) in adult cancer survivors to reflect inherit sex differences in GGT level^26^.

### Definition of ischemic stroke

Incident ischemic stroke was defined as the date of first hospitalization (ICD-10 code: I63) that last at least 48 h. Previous studies using the NHIS-HEALS database have used the diagnosis code along with hospitalization records to identify cerebrovascular disease^27–30^. Among the participants included in the final analytic cohort, follow-up for incident ischemic stroke began on January 1, 2011, and lasted until December 31, 2019. The participants were censored at the first calendar date of incident ischemic stroke, other cerebrovascular disease (ICD-10 codes: I60–I69), death from ischemic stroke or other causes, or end of follow-up, whichever occurred first.

### Covariates

The variables chosen for covariates in this study were obtained from the NHIS-HEALS database. Sociodemographic factors (age, sex, residential area [capital, metropolitan, or rural], health insurance type [employee-insured, self-employed insured], insurance premium [proxy for income status and grouped into quartile]) were derived from health insurance eligibility assessment database. Information on cigarette smoking (never-smoker, past smoker, or current smoker) and alcohol consumption (none, 1–2 times/week, 3–4 times/week, ≥ 5 times/week), physical activity (< 600 metabolic equivalent task [MET]/min, 600–1499 MET/week, ≥ 1500 MET/week), and family history of stroke was obtained through the self-reported questionnaire. Body mass index (BMI), fasting serum glucose (FSG), total cholesterol (TC), aspartate transaminase (AST), and alanine transaminase (ALT) are all anthropometric indicator and biomarkers that were measured in the cohort from the national health screening program. BMI was measured by trained health professionals and calculated by weight in kilograms divided by height in meters squared. Measurement of FSG, TC, AST, and ALT were obtained from biochemical assays from the laboratory test. Aspirin use was defined as 30 or more days of prescription use, and the Charlson comorbidity index (a scoring system to assess the severity of comorbidities) was calculated using medical claims records except for cancer (i.e., cancer without metastasis or metastatic solid tumor). Data pertaining to primary cancer site (head and neck [ICD-10: C00–C14], stomach [ICD-10: C16], colorectal [ICD-10: C18–C20], liver [ICD-10: C22], lung [ICD-10: C34], thyroid [ICD-10: C73], and others) and initial treatment for cancer (surgery only, chemotherapy only, radiotherapy only, combination therapy) were retrieved from the medical claims records between January 1, 2003 and December 31, 2005.

### Statistical analysis

Sociodemographic factors and clinical characteristics of adult cancer survivors at baseline (i.e., prior to the index date, which began on January 1, 2010) according to sex-specific quartiles of GGT level were assessed with mean (standard deviation, SD), number (percentage), and median (interquartile range, IQR) and compared with t-test for continuous variables, chi-square test as well as Kruskal–Wallis test for median values where applicable, respectively. Cumulative incidence function^31^ was used to compare the cumulative incidence of ischemic stroke across the quartiles of GGT levels. To further investigate the association between GGT level and ischemic stroke and adult cancer survivors, we computed hazard ratios (HR) and 95% confidence intervals (CIs) for ischemic stroke according to the quartiles of GGT levels using Cox proportional hazards model^32^. Proportionality assumption of the Cox proportional hazards model was tested with Schoenfeld residual^33^. We developed a minimally adjusted model that adjusted for age and sex only, as well as two multivariable models. Model 1 included adjustments for age, sex, residential area, health insurance type, insurance premium, body mass index, fasting serum glucose, total cholesterol, aspartate aminotransferase, alanine transaminase, cigarette smoking, alcohol consumption, and physical activity. Model 2 further adjusted for family history of stroke, Charlson comorbidity index^34^, and aspirin use, in addition to the variables included in model 1. Model 3 was further adjusted for atrial fibrillation/flutter in addition to the variables included in model 2. In sensitivity analyses, we assessed the risk of ischemic stroke per 10 unit increase in GGT as a continuous variable. Also, we further adjusted for cancer subtypes that are associated with higher level of GGT and risk of stroke (i.e., liver cancer and pancreatic cancer)^35,36^. The minimally adjusted model was used as a baseline for comparison, and the multivariable models were developed to provide a more comprehensive understanding of the various factors that may contribute to stroke risk. These models included a range of demographic, clinical, lifestyle, and health-related variables, and were adjusted for potential confounders. Also, we graphically assessed the association between GGT level and ischemic stroke with restricted cubic spline model, fitted with number of knots selected from Bayesian Information Criterion. Subgroup analyses were conducted to explore whether the association between GGT level and ischemic stroke was consistent across clinically important subgroups. These subgroups were defined by age (< 65 years and ≥ 65 years), sex (male and female), insurance premium (lower half and upper half), and alcohol consumption (drinker and non-drinker). The purpose of these subgroup analyses was to investigate potential effect modification, or whether the association between GGT level and ischemic stroke differed across these important subgroups. Statistical significance was determined using a two-sided probability value of less than 0.05. All statistical analyses were conducted using SAS 7.1 Enterprise Guide (SAS Institute, Cary, NC, USA).

### Ethics declarations

The Institutional Review Board at the National Cancer Center (NCC) approved this study (IRB.: NCC2023-0013). The National Health Insurance Service (NHIS) Big Data Steering Department approved the use of NHIS-Health Screening Cohort (NHIS-HEALS) for this study (NHIS-2022-2-083). As the data used in this study were anonymized, we were exempted from obtaining informed consent from study participants from the Institutional Review Board at the National Cancer Center approved this study (IRB.: NCC2023-0013).

## Results

A total of 3,095 adult cancer survivors (mean age at baseline 60.3 years; range 48–87 years; 63.6% male and 36.4% female) were included in the final analytic cohort. The cohort of survivors in this study had a high prevalence of primary cancers in the stomach, colorectal, and thyroid regions. Furthermore, approximately two thirds of the survivors received more than one type of therapy for cancer, indicating that they may have experienced multiple treatment-related toxicities. Adult cancer survivors with elevated GGT level tended to be younger, male, of lower socioeconomic status (i.e., insurance premium used as a proxy), with higher level of BMI, AST, ALT, current or past smokers, regularly consume alcohol, use aspirin, with more comorbid conditions, with family history of stroke (*P* < 0.05 for all comparisons). The baseline characteristics of the study population are shown in Table 1.

**Table 1:** Sociodemographic and clinical characteristics of adult cancer survivors by sex-specific quartiles of gamma-glutamyl transferase in the National Health Insurance Service-Health Screening Cohort.

Quartiles of GGT^a^	Q1 (n = 688)	Q2 (n = 844)	Q3 (n = 798)	Q4 (n = 765)	*P*-value^b^
Age, years, mean (SD)	61.5 (9.1)	60.7 (9.2)	59.6 (8.7)	59.6 (8.5)	< 0.0001
Sex					< 0.0001
Male	519 (24.6)	571 (67.7)	459 (57.5)	419 (54.8)	
Female	169 (75.4)	273 (32.3)	339 (42.5)	346 (45.2)	
Residential area					0.334
Capital	104 (15.1)	136 (16.1)	139 (17.4)	112 (16.6)	
Metropolitan	463 (67.3)	588 (69.7)	535 (67.1)	543 (70.9)	
Rural	121 (17.6)	120 (14.2)	124 (15.5)	110 (13.5)	
Health insurance type					0.106
Employee insured	559 (81.3)	692 (82.0)	624 (78.2)	588 (76.9)	
Self-employed insured	129 (18.7)	152 (18.0)	174 (21.8)	177 (23.1)	
Insurance premium					0.008
Q1 (lowest)	266 (38.6)	358 (42.4)	336 (42.1)	315 (41.2)	
Q2	114 (16.6)	169 (20.0)	147 (18.4)	127 (16.6)	
Q3	204 (29.7)	200 (23.7)	181 (22.7)	226 (29.5)	
Q4 (highest)	104 (15.1)	117 (13.9)	134 (16.8)	97 (12.7)	
BMI, kg/m^2^, mean (SD)	22.3 (2.7)	23.0 (3.0)	24.0 (3.0)	24.1 (3.2)	< 0.0001
FSG, mg/dL, mean (SD)	98.9 (21.9)	100.1 (21.2)	102.1 (26.2)	104.2 (30.9)	< 0.0001
TC, mg/dL, mean (SD)	184.0 (31.3)	190.9 (32.8)	196.9 (34.9)	192.6 (37.3)	< 0.0001
AST, IU/L, median (IQR)	23.0 (20.0–28.0)	24.0 (20.0–29.0)	24.0 (20.0–30.0)	29.0 (24.0–39.0)	< 0.0001
ALT, IU/L, median (IQR)	18.0 (14.0–23.0)	20.0 (15.0–26.0)	22.0 (18.0–29.0)	27.0 (20.0–39.0)	< 0.0001
GGT, IU/L, median (IQR),	15.0 (11.0–18.0)	22.0 (14.0–26.0)	32.0 (19.0–40.0)	62.0 (35.0–93.0)	< 0.0001
Cigarette smoking					< 0.0001
Never-smoker	379 (55.1)	487 (57.7)	484 (60.7)	474 (61.9)	
Past smoker	235 (34.2)	273 (32.4)	212 (26.6)	149 (19.5)	
Current smoker	74 (10.7)	84 (9.9)	102 (12.7)	142 (18.6)	
Alcohol consumption					< 0.0001
None	522 (75.9)	596 (70.6)	522 (65.4)	429 (56.1)	
1–2 times/week	123 (17.8)	173 (20.5)	193 (24.2)	153 (20.0)	
3–4 times/week	18 (2.6)	42 (4.9)	57 (7.1)	111 (14.5)	
≥ 5 times/week	25 (3.7)	33 (4.0)	26 (3.3)	72 (9.4)	
Physical activity					0.966
< 600 MET/week	214 (31.1)	257 (30.4)	359 (31.1)	237 (30.9)	
600–1499 MET/week	335 (48.7)	409 (48.5)	385 (48.3)	383 (50.0)	
≥ 1500 MET/week	139 (20.2)	178 (21.1)	165 (20.6)	145 (19.1)	
Aspirin use^c^	36 (5.2)	66 (7.8)	95 (11.9)	78 (10.2)	< 0.0001
Atrial fibrillation/flutter	9 (1.3)	17 (2.0)	6 (0.8)	12 (1.6)	0.183
Charlson comorbidity index					< 0.001
0	69 (10.0)	62 (7.4)	58 (7.3)	44 (5.8)	
1	194 (28.2)	239 (28.3)	221 (27.7)	192 (25.1)	
2	235 (34.2)	279 (33.1)	235 (29.5)	231 (30.2)	
≥ 3	190 (27.6)	264 (31.2)	284 (35.5)	298 (38.9)	
Family history of stroke	32 (4.7)	36 (4.3)	72 (9.0)	41 (6.7)	< 0.001
Primary cancer site					
Head and neck	13 (1.9)	19 (2.3)	26 (3.3)	23 (3.0)	0.306
Stomach	303 (44.0)	248 (29.4)	171 (21.4)	111 (14.5)	< 0.0001
Colorectal	121 (17.6)	163 (19.3)	135 (16.9)	135 (17.7)	0.626
Liver	16 (2.3)	41 (4.9)	54 (6.8)	113 (14.8)	< 0.0001
Pancreas	1 (0.2)	6 (0.7)	8 (1.0)	14 (1.8)	0.008
Lung	37 (5.4)	48 (5.7)	58 (7.3)	35 (4.6)	0.137
Thyroid	78 (11.3)	130 (15.4)	172 (21.6)	148 (19.4)	< 0.0001
Others	120 (17.5)	195 (23.0)	182 (22.7)	200 (26.0)	0.015
Initial treatment for cancer					
Surgery (only)	69 (10.1)	104 (12.3)	89 (11.2)	92 (12.0)	0.508
Chemotherapy (only)	62 (9.0)	72 (8.5)	49 (6.1)	40 (5.2)	0.009
Radiotherapy (only)	75 (10.9)	76 (9.0)	94 (11.8)	75 (9.8)	0.275
Combination^d^	482 (70.0)	592 (70.2)	566 (70.9)	558 (73.0)	0.323

Data above are presented as n(%) unless otherwise specified.

*Q* quartile, *SD* standard deviation, *GGT* gamma-glutamyl transferase, *IQR* interquartile range, *BMI* body mass index, *FSG* fasting serum glucose, *TC* total cholesterol, *AST* aspartate aminotransferase, *ALT* alanine transaminase, *MET* metabolic equivalent task.

^a^Sex-specific quartiles (male: ≤ 20, 21–30, 31–52, ≥ 54 IU/L; female: ≤ 11, 12–15, 16–22, ≥ 23 IU/L) for GGT level.

^b^Based on Chi-square test (categorical variables), t-test (continuous variable), Kruskal–Wallis Test (median values).

^c^Defined as more than 30 days of prescription.

^d^Receiving more than one type of treatment.

Over a median follow-up of 8.9 years from the index date, 80 (2.58%) incident ischemic stroke cases were documented in 3095 adult cancer survivors. The analysis showed that there was a trend of increasing risk for ischemic stroke with higher GGT quartiles. The multivariable adjusted HRs for ischemic stroke were 1.56 (95% Cis 0.75–3.26), 2.36 (95% Cis 1.12–4.99), and 2.40 (95% Cis 1.05–5.46) in the quartile 2, quartile 3, and quartile 4 as compared with those with the lowest quartile of GGT (*P*_*trend*_ = 0.013) (Table 2). In multivariable-adjusted analyses, a 10-unit increase in GGT was significantly associated with risk of ischemic stroke (Supplementary Table S1). Further adjusting for cancer subtypes associated with GGT level and stroke risk did not significantly alter the association between GGT level and ischemic stroke (Supplementary Table S2).

**Table 2:** Hazard ratio of ischemic stroke by sex-specific quartiles of gamma-glutamyl transferase in adult cancer survivors in the National Health Insurance Service-Health Screening Cohort.

Quartiles of GGT^a^	Q1 (n = 688)	Q2 (n = 844)	Q3 (n = 798)	Q4 (n = 765)	*P*_trend_
Event, no	11	22	27	20	
Person-years	5679	6992	6493	6062	
Age- and sex-adjusted HR (95% CI)	1.00 [reference]	1.80 (0.87–3.71)	2.86** (1.41–5.80)	2.43* (1.15–5.12)	0.007
Multivariable-adjusted HR (95% CI), model 1^b^	1.00 [reference]	1.67 (0.80–3.49)	2.63* (1.26–5.51)	2.50* (1.10–5.68)	0.013
Multivariable-adjusted HR (95% CI), model 2^c^	1.00 [reference]	1.59 (0.76–3.32)	2.42* (1.14–5.12)	2.41* (1.06–5.49)	0.019
Multivariable-adjusted HR (95% CI), model 3^d^	1.00 [reference]	1.56 (0.75–3.26)	2.36* (1.12–4.99)	2.40* (1.05–5.46)	0.013

*Q* quartile, *GGT* gamma-glutamyl transferase, *IQR* interquartile range, *No* number, *HR* hazard ratio, *CI* confidence interval.

^a^Sex-specific quartiles (male: ≤ 20, 21–30, 31–52, ≥ 54 IU/L; female: ≤ 11, 12–15, 16–22, ≥ 23 IU/L) for GGT level.

^b^Cox proportional hazards model adjusted for age, sex, residential area, health insurance type, insurance premium, body mass index, fasting serum glucose, total cholesterol, aspartate aminotransferase, alanine transaminase, cigarette smoking, alcohol consumption, physical activity.

^c^Adjusted for family history of stroke, Charlson comorbidity index, and aspirin use in addition to the variables included in model 1.

^d^Adjusted for presence of atrial fibrillation/flutter in addition to the variables included in model 2.

*p < 0.05, **p < 0.01.

**Figure 2:**
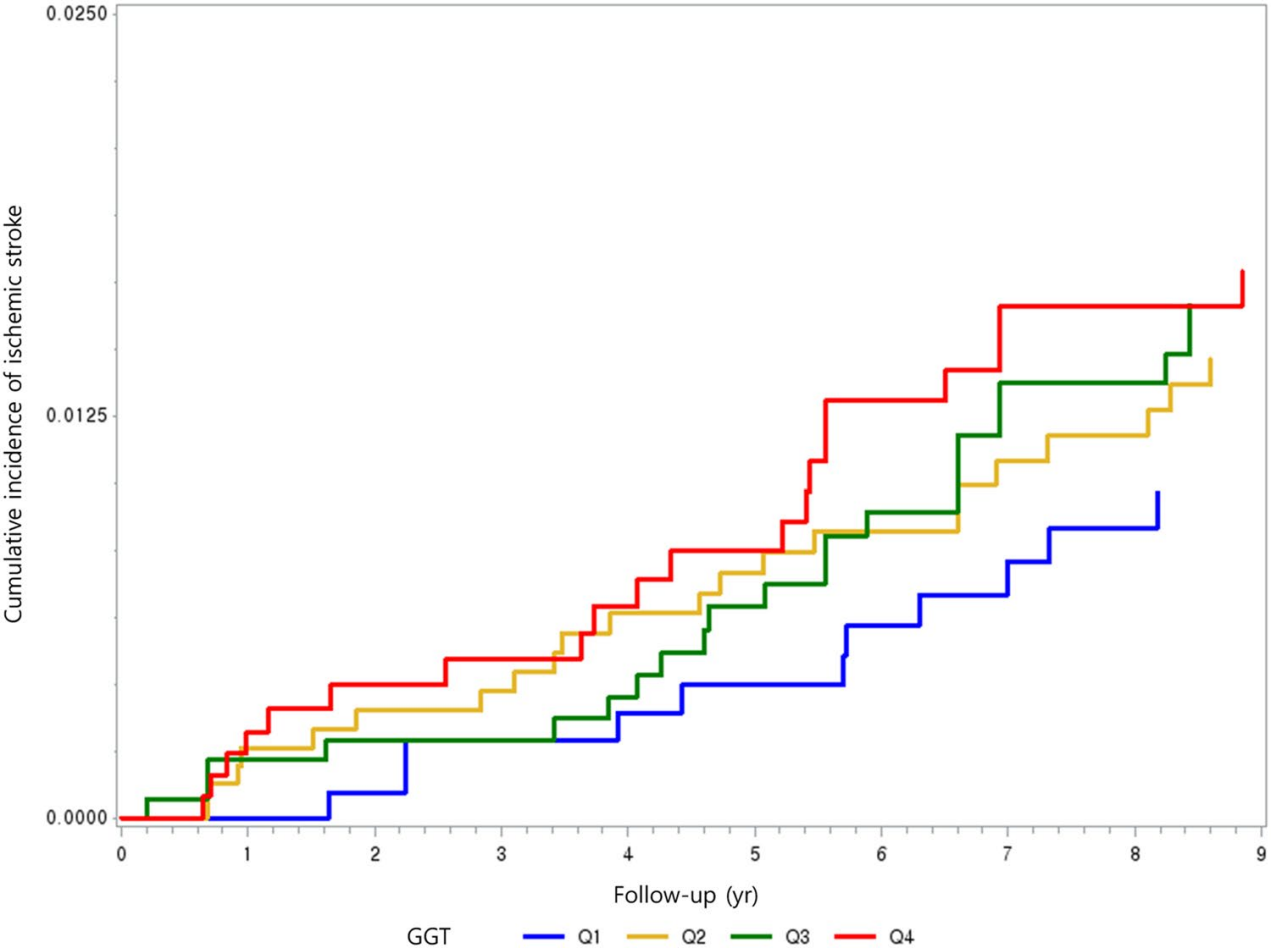
HR with 95% CIs (GGT 10.0 IU/L as reference) for incident ischemic stroke using baseline data on GGT from 3,095 adult cancer survivors who received national health screening between 2009 and 2010 and followed up from 2011 to 2019. Restricted cubic spline model was estimated with Cox proportional hazards regression models (adjusted for age, sex, residential area, health insurance type, insurance premium, body mass index, fasting serum glucose, total cholesterol, aspartate aminotransferase, alanine transaminase, cigarette smoking, alcohol consumption, physical activity, family history of stroke, Charlson comorbidity index, and aspirin use). *HR* hazard ratio, *CI* confidence intervals, *GGT* gamma-glutamyl transferase. Four knots were placed at the 5th, 33rd, 67th, and 95th percentiles.

**Figure 3:**
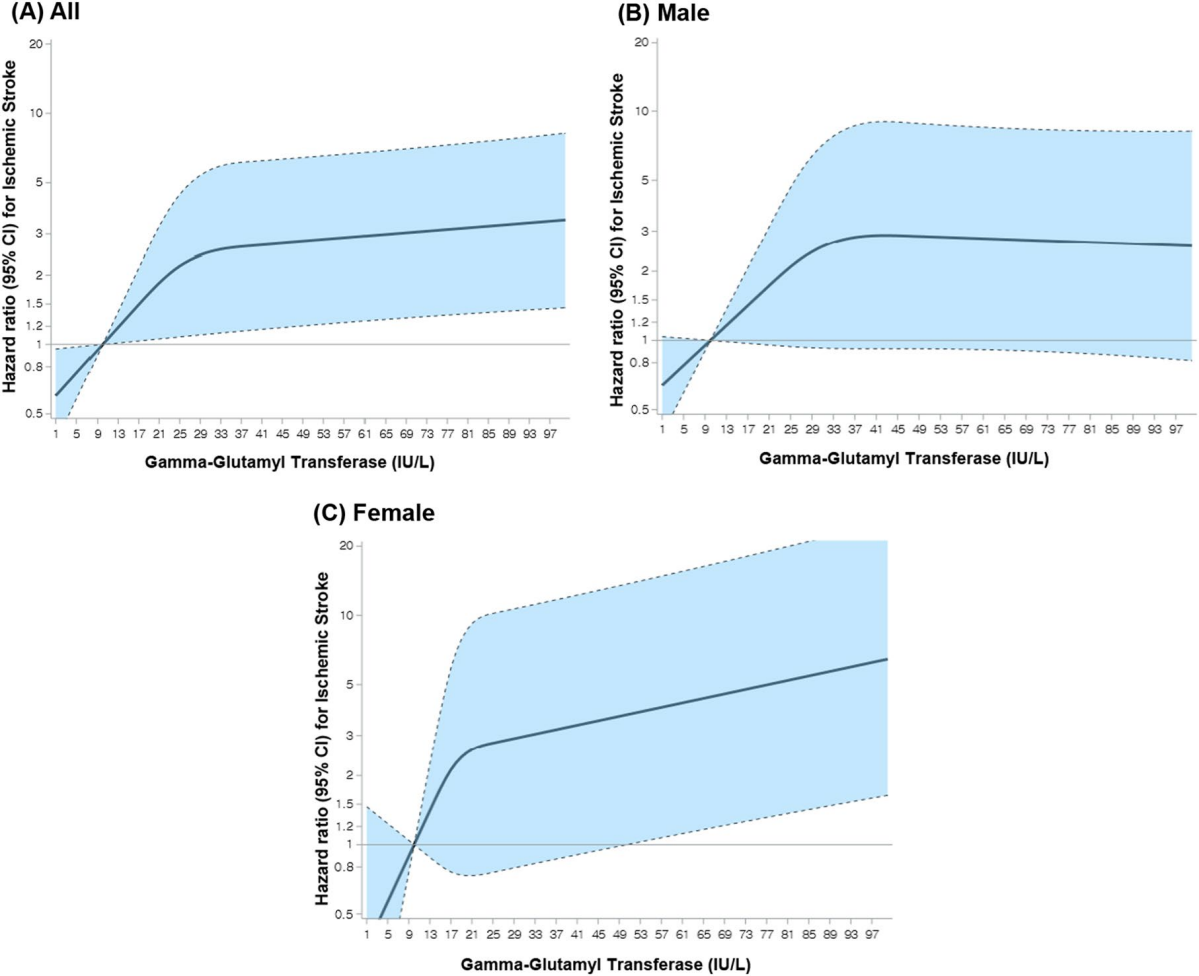
Cumulative incidence of ischemic stroke according to quartiles of gamma-glutamyl transferase level among adult cancer survivors. *GGT* gamma-glutamyl transferase, *Q* quartiles.

**Figure 4:**
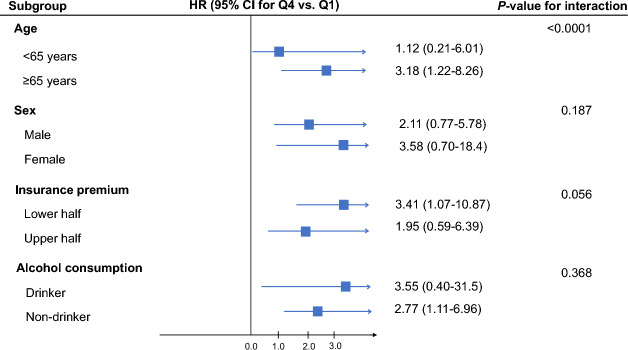
Subgroup plot of hazard ratios for incident ischemic stroke in adult cancer survivors stratified by age, sex, insurance premium, and alcohol consumption. Hazard ratio and 95% confidence intervals were derived from Cox proportional hazards model adjusted for age, sex, residential area, health insurance type, insurance premium, body mass index, fasting serum glucose, total cholesterol, aspartate aminotransferase, alanine transaminase, cigarette smoking, alcohol consumption, physical activity, family history of stroke, Charlson comorbidity index, and aspirin use (except for the variable in each subgroup). *HR* hazard ratio, *CI* confidence intervals, *Q* quartile.

The cumulative incidence function showed that the highest incidence of ischemic stroke was observed in the GGT quartile 4, while the lowest incidence was observed in the GGT quartile 1 (Fig. 2). The dose–response association between GGT level and the risk of ischemic stroke is illustrated in Fig. 3. This relationship between GGT and the incidence of ischemic stroke was modeled with multivariable-adjusted spline regression model (Model 2), with a reference point set to 10 IU/L. The graphs shows that the risk of ischemic stroke increased proportionally with GGT up to the point where GGT exceeded approximately 50 IU/L. This indicates that there may be a threshold above which higher GGT level does not necessarily further increase the risk of ischemic stroke in this cohort of cancer survivors. Figure 4 lists a summary of the results from the subgroup analyses conducted to investigate the association between GGT level and the risk of incident ischemic stroke stratified by age, sex, insurance premium, and alcohol consumption. Higher risk of ischemic stroke was found in adult cancer survivors aged 65 years and older (HR = 3.18; 95% CIs 1.22–8.26) was compared to those under the age of 65 (HR = 1.12; 95% CIs 0.21–6.01) (*P for interaction* < 0.0001). Similar association was observed in female (HR = 3.58; 95% CIs 0.70–18.4) compared to male (HR = 2.11; 95% CIs 0.77–5.78), among those with lower socioeconomic status (HR = 3.41; 95% CIs 1.07–10.87) compared to higher socioeconomic status (HR = 1.95; 95% CIs 0.59–6.39), and among alcohol drinkers (HR = 3.55; 95% CIs 0.40–31.5) compared to alcohol non-drinkers (HR = 2.77; 95% CIs 1.11–6.96). However, no effect modification was observed by sex, insurance premium, and alcohol consumption (*P for interaction* > 0.05).

## Discussion

In this population-based cohort study of adult cancer survivors, we observed an increased risk of ischemic stroke among those with elevated GGT level. Compared to those with normal range of GGT, adult cancer survivors with elevated GGT level had a 2.4-fold increased risk of ischemic stroke, which was independent of alcohol consumption status. We also observed that beyond a particular elevated level of GGT (approximately 50 IU/L), the risk of ischemic stroke did not significantly change with further increases in GGT level. Stratified analyses also showed that the association of high GGT level with increased risk of ischemic stroke was not modified by sex, insurance premium, and alcohol consumption.

In clinical practice, GGT has traditionally served as a proxy marker for alcohol consumption, due to the increased levels in individuals who consume alcohol on regular basis. However, its role has evolved to encompass the evaluation of hepatic inflammation, fatty liver disease, and hepatitis. Thus, GGT has emerged as a crucial diagnostic tool for assessing liver health and identifying liver-related conditions due to its widespread availability and broad applicability. Moreover, evidence from observational studies in the past decades have showed association of elevated GGT levels with a higher stroke incidence and mortality^37–41^. A meta-analysis of 10 prospective cohort studies including 926,497 participants with 5707 cases showed a pooled hazard ratio of 1.28 (95% CI 1.16–1.43; *I*^2^ = 74.5%) for risk of incident stroke among those with high GGT level as compared to those with low GGT level^13^. Only two studies included in the meta-analyses were of Asian descendants who were predominately male participants with relatively higher cut-off points for GGT level compared to the participants in the North American or European cohorts included in the pooled analysis^42,43^.

Also, a retrospective analysis of the NHIS-National Sample Cohort reported a 45% increased risk of ischemic stroke among 456,100 individuals with high GGT level as compared to those with low GGT level stratified by sex-specific quartiles^25^. While these findings establish the evidence on GGT as a biomarker for increased cardiovascular risk, none have specifically addressed this association in population with history of cancer. Adult cancer survivors are at increased risk of stroke attributable to treatment related factors such as chemotherapy (e.g., anthracyclines and monoclonal antibodies) and immunotherapy (e.g., selective estrogen receptor modulators and aromatase inhibitors) along with shared lifestyle-related risk factors (e.g., obesity, cigarette smoking, hypertension) between cancer and ischemic stroke^5^. Our study provides additional evidence supporting the need for additional clinical attention for stroke prevention among adult cancer survivors with elevated GGT level.

Several biological mechanisms may explain the increased stroke risk associated with GGT level found in our study. As opposed to extracellular GGT, the exact mechanisms underlying how serum GGT may contribute to development of ischemic stroke in adult cancer survivors is not entirely clear. However, there is a biologically plausible hypothesis regarding the role of elevated serum GGT level on oxidative stress and inflammatory pathway in stroke pathophysiology^44^. Experimental evidence suggests that GGT may promote the production of reactive oxygen species that could lead to depletion of intracellular glutathione, which could further exacerbate oxidative stress^45^. Prolonged oxidative stress is known to induce atherosclerosis, which is one of the major contributors to onset of ischemic stroke^46,47^. Thus, adult cancer survivors with elevated level of GGT under conditions of high oxidative stress are more susceptible to production of reactive oxygen species that could eventually contribute to higher risk of ischemic stroke as compared to those with normal level of GGT. Additionally, in the context of ischemic stroke risk, it is plausible that there exists a critical threshold of GGT levels beyond which the magnitude of ischemic stroke risk does not linearly escalate. Hypothetically, once GGT levels surpass this critical threshold, any subsequent elevation may only lead to marginal increments in ischemic stroke risk. Given the observational nature of our study, further studies with detailed data on biomarkers for oxidative stress and underlying biological mechanisms for the threshold for GGT level are needed.

### Study strengths and limitations

Strengths of our study are identifying adult cancer survivors with validated information on GGT level and stroke outcome from a population-based database, which also included assessment of lifestyle behavior and sociodemographic factors used for adjustment in the analyses. In addition, the utilization of regularly updated claims data under a single insurer system enabled the identification and tracking of ischemic stroke events that occurred during the follow-up period. However, some limitations of the present study should be noted. First, the study population consisted solely of adult cancer survivors of Korean population. Therefore, the generalizability to other ethnic groups may be limited considering the differences in primary cancer sites and treatment strategies in addition to ethnic differences. Second, self-reported health behavior (i.e., cigarette smoking, physical activity, and alcohol consumption) may have led to underreporting of smoking and drinking habits and potentially generated biased results in the adjusted analyses with Cox proportional hazards model. Third, data on GGT level during the follow-up period was not available. Thus, future investigations are warranted to address whether changes in GGT level significantly alter the association of baseline GGT level and ischemic stroke risk found in this study. Fourth, statistical significance for the association between GGT level and ischemic stroke was drastically attenuated in subgroup analyses partially owing to a relatively small number of outcome events in each subgroup. Lastly, because our dataset of adult cancer survivor population was derived from individuals who initially received national health screening rather than cancer diagnosis, the population size was relatively smaller than other nationwide cohort studies. Therefore, the generalizability of the findings in this study should be tested in larger cohort of adult cancer survivors with validated GGT level and ischemic stroke events in the future.

## Conclusions

Adult cancer survivors with elevated level of GGT, regardless of alcohol consumption status, are at increased risk of ischemic stroke compared to those with normal level of GGT. Findings of our study suggest that adult cancer survivors with elevated GGT level may need additional clinical attention for management of future ischemic stroke risk. While GGT has been considered solely as a surrogate marker of alcohol consumption, our study suggests that GGT may also be used as a risk factor for assessing stroke risk along with other traditional risk factors in the cancer survivor population.

### Supplementary Information

Supplementary Tables.

## Data Availability

The study utilized datasets from the secure server of the National Health Insurance Service Big Data Steering Department. All analyses were conducted with the anonymized dataset provided to the authorized individuals. To learn more about the process of accessing these datasets, please refer to the instructions provided at https://nhiss.nhis.or.kr/.
