# Supplementary material for: Increased risk of ischemic stroke associated with elevated gamma-glutamyl transferase level in adult cancer survivors: a population-based cohort study
Source: Sci Rep. 2023 Oct 6;13:16837. doi: 10.1038/s41598-023-43839-8 (PMC10558526; doi:10.1038/s41598-023-43839-8)
Supplement: Supplementary file 1 — Supplementary Tables. [file 41598_2023_43839_MOESM1_ESM.docx]

**Supplementary Material**

**Supplementary Table 1.** Risk of ischemic stroke per 10 unit increase of gamma-glutamyl transferase in adult cancer survivors in the National Health Insurance Service-Health Screening Cohort

**Supplementary Table 2.** Hazard ratio of ischemic stroke by sex-specific quartiles of gamma-glutamyl transferase by additionally adjusting for liver or pancreatic cancer among adult cancer survivors in the National Health Insurance Service-Health Screening Cohort

**Supplementary Table 1.** Risk of ischemic stroke per 10 unit increase of gamma-glutamyl transferase in adult cancer survivors in the National Health Insurance Service-Health Screening Cohort

|  | **Parameter Estimate** | **Standard Error** | **Hazard Ratio** | **95% Confidence intervals** | ***P*-value** |
| --- | --- | --- | --- | --- | --- |
| Age-and sex- adjusted HR (95% CI) | 0.04277 | 0.01860 | 1.044 | 1.006-1.082 | 0.0215 |
| Multivariable-adjusted HR  (95% CI), Model 1^a^ | 0.04925 | 0.02197 | 1.050 | 1.006-1.097 | 0.0250 |
| Multivariable-adjusted HR  (95% CI), Model 2^b^ | 0.05286 | 0.02246 | 1.054 | 1.009-1.102 | 0.0186 |
| Multivariable-adjusted HR  (95% CI), Model 3^c^ | 0.05529 | 0.02220 | 1.057 | 1.012-1.104 | 0.0128 |
| Multivariable-adjusted HR  (95% CI), Model 4^c^ | 0.00553 | 0.00221 | 1.057 | 1.012-1.104 | 0.0124 |

^a^Cox proportional hazards model adjusted for age, sex, residential area, health insurance type, insurance premium, body mass index, fasting serum glucose, total cholesterol, aspartate aminotransferase, alanine transaminase, cigarette smoking, alcohol consumption, physical activity

^b^Adjusted for family history of stroke, Charlson comorbidity index, and aspirin use in addition to the variables included in Model 1

^c^Adjusted for presence of atrial fibrillation/flutter in addition to the variables included in Model 2

^d^Adjusted for liver or pancreatic cancer in addition to the variables included in Model 3

**Supplementary Table 2.** Hazard ratio of ischemic stroke by sex-specific quartiles of gamma-glutamyl transferase by additionally adjusting for liver or pancreatic cancer among adult cancer survivors in the National Health Insurance Service-Health Screening Cohort

| **Quartiles of GGT^a^** | **Q1**  **(n=688)** | **Q2**  **(n=844)** | **Q3**  **(n=798)** | **Q4**  **(n=765)** | ***P*_trend_** |
| --- | --- | --- | --- | --- | --- |
| Event, No. | 11 | 22 | 27 | 20 |  |
| Person-years | 5,679 | 6,992 | 6,493 | 6,062 |  |
| Multivariable-adjusted HR  (95% CI)^b^ | 1.00  [Reference] | 1.57  (0.75-3.28) | 2.39^*^  (1.13-5.05) | 2.45^*^  (1.06-5.63) | 0.012 |

^a^Sex-specific quartiles (male: ≤20, 21-30, 31-52, ≥54 IU/L; female: ≤11, 12-15, 16-22, ≥23 IU/L) for GGT level

^b^Cox proportional hazards model adjusted for age, sex, residential area, health insurance type, insurance premium, body mass index, fasting serum glucose, total cholesterol, aspartate aminotransferase, alanine transaminase, cigarette smoking, alcohol consumption, physical activity, family history of stroke, Charlson comorbidity index, and aspirin use, presence of atrial fibrillation/flutter, presence of liver or pancreatic cancer

Abbreviations: Q, quartile; GGT, gamma-glutamyl transferase; IQR, interquartile range; No, number; HR, hazard ratio; CI, confidence interval

**p*<0.05, ***p*<0.01
